# Hospital intervention volume affects outcomes of emergency transcatheter aortic valve implantations in Germany

**DOI:** 10.1038/s41598-022-20336-y

**Published:** 2022-10-19

**Authors:** Vera Oettinger, Adrian Heidenreich, Klaus Kaier, Manfred Zehender, Christoph Bode, Daniel Duerschmied, Constantin von zur Mühlen, Dirk Westermann, Peter Stachon

**Affiliations:** 1 Department of Cardiology and Angiology, University Heart Center, Faculty of Medicine, Medical Center, University of Freiburg, Hugstetter Str. 55, 79106 Freiburg, Germany; 2 Center for Big Data Analysis in Cardiology (CeBAC), Department of Cardiology and Angiology, University Heart Center, Faculty of Medicine, Medical Center, University of Freiburg, Freiburg, Germany; 3 Institute of Medical Biometry and Statistics, Faculty of Medicine and Medical Center, University of Freiburg, Freiburg, Germany; 4 Department of Cardiology, Angiology, Haemostaseology and Medical Intensive Care, University Medical Centre Mannheim, Medical Faculty Mannheim, Heidelberg University, Mannheim, Germany; 5 European Center for AngioScience (ECAS) and German Center for Cardiovascular Research (DZHK) Partner Site Heidelberg/Mannheim, Mannheim, Germany

**Keywords:** Cardiology, Interventional cardiology, Health care economics, Outcomes research

## Abstract

The literature has shown an inverse volume-outcome relationship for transcatheter aortic valve implantation (TAVI). However, little is known about emergency admissions in Germany. Using German national electronic health records, we identified all isolated balloon-expandable and self-expanding transfemoral TAVI in 2018. The focus was on those patients with emergency admission. 17,295 patients were treated with TAVI, including 1682 emergency cases. 49.2% of the emergency admissions were female, the mean age was 81.2 years and the logistic EuroSCORE was 23.3%. The percentage of emergency cases was higher in lower volume than in higher volume centers (*p* < 0.001): In detail, centers performing < 50 TAVI showed an emergency admission rate of ~ 15%, those with > 200 TAVI a rate of ~ 11%. After propensity score adjustment, analyzing the outcomes for an increase in volume per 10 emergency admissions, higher volume centers showed significantly better outcomes regarding in-hospital mortality (OR = 0.872, *p* = 0.043), major bleeding (OR = 0.772, *p* = 0.001), stroke (OR = 0.816, *p* = 0.044), mechanical ventilation > 48 h (OR = 0.749, *p* = 0.001), length of hospital stay (risk adjusted difference in days of hospitalization per 10 emergency admissions: − 1.01 days, *p* < 0.001), and reimbursement (risk adjusted difference in reimbursement per 10 emergency admissions: -€314.89, *p* < 0.001). Results were not significant for acute kidney injury (OR = 0.951, *p* = 0.104), postoperative delirium (OR = 0.975, *p* = 0.480), and permanent pacemaker implantation (OR = 1.010, *p* = 0.732). In conclusion, regarding transfemoral TAVI, the percentage of emergency cases was higher in lower volume centers in Germany. However, higher volume centers show significantly better outcomes for in-hospital mortality and complication rates as well as resource utilization parameters.

## Introduction

Transcatheter aortic valve implantation (TAVI) has developed rapidly over the last 20 years^1–3^. The indication, which was initially limited to patients with isolated aortic valve stenosis and a very high operative risk, was increasingly extended, since even patients with a low operative risk benefit in particular from transfemoral TAVI^4–8^. Meanwhile, TAVI is the most commonly used treatment for aortic valve stenosis in the United States^9^ and Germany^2,7^. Furthermore, literature showed an inverse volume-outcome relationship for TAVI in general, i.e. better outcomes with increasing annual hospital case numbers^10–15^, but little is known about emergency cases.

Bansal et al.^16^ examine in a recent study the influence of annual hospital volumes on the use and the outcome of urgent and emergent TAVI in the United States. They show that higher volume centers perform more urgent and emergent TAVI. Moreover, the authors report on better results in centers with higher volume of urgent and emergent TAVI procedures for in-hospital mortality, stoke, acute kidney injury, vascular complications, and length of stay. However, the relationship between center volume and use or outcome in the case of TAVI with emergency admission has not yet been investigated in Germany. This is particularly interesting because Germany has a different medical care structure than the United States.

The present study aims to examine the effect of annual hospital volume on treatment with TAVI in case of emergency. Therefore, we performed an analysis of all 17,295 patients treated with balloon-expandable or self-expanding transfemoral TAVI in Germany in 2018 with a focus on those 1682 patients with emergency admission. The distribution of emergency interventions across centers of different sizes as well as a possible volume-outcome relationship with regard to in-hospital mortality and other outcomes including resource utilization is to be investigated.

## Material and methods

Since 2005, data on all hospitalizations in Germany have been available for scientific use via the Diagnosis Related Groups (DRG) statistics collected by the Research Data Center of the Federal Bureau of Statistics (DESTATIS). These hospitalization data, including diagnoses and procedures, are a valuable source of representative nationwide data on the in-hospital treatment of patients. This database represents a virtually complete collection of all hospitalizations in German hospitals that are reimbursed according to the Diagnosis Related Groups system. From this database, we extracted data on all isolated balloon-expandable or self-expanding transfemoral TAVI procedures conducted in 2018. As described previously, patients with a baseline diagnosis of pure aortic regurgitation (main or secondary diagnosis other than I35.0, I35.2, I06.0, I06.2) and those with concomitant cardiac surgery or percutaneous coronary intervention were not included in this analysis^2^. A complete list of procedure codes may be found in a previous article^17^. Furthermore, those patients with emergency admission were extracted. Emergency admission was a predefined code that was made available for scientific analysis by DESTATIS after transmission by the hospitals.

### Endpoints

The analysis focused on eight different end points: in-hospital mortality, bleeding events, stroke, acute kidney injury, postoperative delirium, permanent pacemaker implantation, mechanical ventilation exceeding 48 h, length of hospital stay, and reimbursement. Stroke and acute kidney injury were defined using ICD, Tenth Revision (ICD-10) codes (secondary diagnosis I63* or I64 and N17*, respectively). Bleeding was defined as requiring a transfusion of > 5 units of red blood cells and identified using the German Operation and Procedure Classification (OPS) codes (8–800.c1 to 8–800.cr). In-hospital mortality, length of mechanical ventilation, and length of hospital stay were part of DESTATIS’ main set of variables. For all other comorbidities, the existing anamnestic or acute distinctive codes were used (we have discussed OPS and ICD codes in detail previously^2^).

For calculation of the estimated logistic EuroSCORE (European System for Cardiac Operative Risk Evaluation), we were able to populate all fields except for critical preoperative state and left ventricular function. In these, we assumed an inconspicuous state (i.e. no critical preoperative state and no left ventricular dysfunction) and thus calculated a best-case scenario.

### Statistical analysis

In a previous study, Reinöhl et al.^2^ identified 20 baseline patient characteristics to describe risk profiles between procedural groups. Since patients were not randomized, potential confounding factors were taken into account using the propensity score methods. In detail, the propensity score was used for adjustment. The propensity score was estimated using a multivariable linear regression model, with the number of emergency TAVI cases as the dependent variable and all of the baseline characteristics listed in Table 1 as independent variables. Then, propensity score adjustment was applied with the number of emergency TAVI cases and the propensity score as continuous covariates. Hereby, logistic or linear regression models were used as appropriate. To account for the correlation of error terms of patients treated in the same hospital, a random intercept was included at the center level. Based on these eight risk-adjusted logistic or linear regression analyses, predicted probabilities (or means) were calculated using marginal standardization and visualized across certain volume categories^18^.

**Table 1:** Baseline characteristics of patients with balloon-expandable or self-expanding TAVI in Germany in 2018.

N	All patients		Emergency patients		*p *value
	17,295		1682		
Self-expanding instead of balloon-expandable	57.14%		57.97%		0.512
Logistic EuroSCORE, mean / SD	13.46	9.84	23.29	13.53	< 0.001
Age in years, mean / SD	81.11	6.08	81.24	6.71	0.407
Female %	50.90%		49.23%		0.190
NYHA II, %	13.21%		8.56%		< 0.001
NYHA III or IV, %	52.14%		59.10%		< 0.001
CAD, %	50.92%		52.14%		0.338
Arterial hypertension, %	63.54%		62.37%		0.341
Previous MI within 4 months, %	1.60%		2.32%		0.027
Previous MI within 1 year, %	0.71%		0.77%		0.775
Previous MI after 1 year, %	4.21%		5.83%		0.002
Previous CABG, %	8.30%		9.33%		0.143
Previous cardiac surgery, %	12.83%		15.76%		< 0.001
Peripheral vascular disease, %	8.52%		9.39%		0.224
Carotid disease, %	6.55%		5.82%		0.253
COPD, %	11.67%		13.50%		0.027
Pulmonary hypertension, %	21.07%		23.84%		0.008
Renal disease, GFR < 15 ml/min, %	2.31%		3.21%		0.021
Renal disease, GFR < 30 ml/min, %	4.27%		6.06%		< 0.001
Atrial fibrillation, %	45.19%		51.55%		< 0.001
Diabetes mellitus, %	32.05%		32.05%		0.997
Emergency, %	9.73%		100.00%		–
Number of cases per center, mean / SD	287.66	138.15	36.29	24.01	–

CABG: coronary artery bypass graft; CAD: coronary artery disease; COPD: chronic obstructive pulmonary disease; EuroSCORE: European System for Cardiac Operative Risk Evaluation; GFR: glomerular filtration rate; MI: myocardial infarction; N: number of procedures; NYHA: New York Heart Association; SD: standard deviation.

p-values based on chi-square test or t-test, as appropriate.

No imputation for missing values could be conducted due to the absence of codes indicating that data were missing. If the patient’s electronic health record did not include information on a clinical characteristic, it was assumed that that characteristic was not present. Furthermore, no adjustment for multiple testing was carried out. Thus, p-values may not be interpreted as confirmatory but are descriptive in nature and inferences drawn from the 95% confidence intervals may not be reproducible.

All analyses were performed with Stata 16 (StataCorp, College Station, Texas, USA).

### Ethics approval and informed consent

Our study did not involve direct access by the investigators to data on individual patients but only access to summary results provided by DESTATIS. Therefore, approval by an ethics committee and informed consent were determined not to be required, in accordance with German law. All summary results were anonymized by DESTATIS. In practice, this means that any information allowing the drawing of conclusions about a single patient or a specific hospital was censored by DESTATIS to guarantee data protection. Moreover, in order to prevent the possibility to draw conclusions to a single hospital the data are verified and situationally censored by DESTATIS in those cases. All methods were carried out in accordance with relevant guidelines and regulations.

## Results

### Baseline characteristics

A total of 17,295 patients were treated with balloon-expandable or self-expanding transfemoral TAVI in Germany in 2018 (Table 1). 1682 of these had an emergency admission. Age was comparable between all patients and emergency admissions with 81.11 vs 81.24 years (*p* = 0.407), which also applies to female sex with 50.90 vs 49.23% (*p* = 0.190). However, logistic EuroSCORE differed distinctly with 13.46% in overall population vs 23.29% in emergency admissions (*p* < 0.001). Also, emergency patients had relatively more higher grade heart failure (all vs emergency patients: NYHA II 13.21 vs 8.56%, *p* < 0.001; NYHA III/IV 52.14 vs 59.10%, *p* < 0.001) as well as more pre-existing diseases examined in baseline characteristics like previous cardiac surgery, pulmonary hypertension, higher grade renal disease or atrial fibrillation.

### Unadjusted in-hospital outcomes

In emergency cases the unadjusted in-hospital mortality was 3.57%, major bleeding 3.27%, stroke 1.90%, acute kidney injury 15.04%, delirium 10.11%, permanent pacemaker implantation 13.85%, mechanical ventilation > 48 h 2.00%, mean length of hospital stay 17.75 days, and mean reimbursement €29,917 (Table 2). Compared to all TAVI procedures, emergency patients had higher or roughly the same rates for almost all unadjusted outcomes, i.e. in-hospital mortality (*p* < 0.001) and complication rates (major bleeding *p* = 0.013, acute kidney injury *p* < 0.001, delirium *p* = 0.017) as well as resource utilization parameters (length of hospital stay *p* < 0.001, reimbursement *p* < 0.001).

**Table 2:** Unadjusted in-hospital outcomes of patients with balloon-expandable or self-expanding TAVI in Germany in 2018.

N	All patients		Emergency patients		*p *value
	17,295		1682		
In-hospital mortality, %	2.25%		3.57%		< 0.001
Bleeding > 5 units, %	2.30%		3.27%		0.013
Stroke, %	1.98%		1.90%		0.833
Acute kidney injury, %	8.41%		15.04%		< 0.001
Delirium, %	8.40%		10.11%		0.017
Permanent pacemaker implantation, %	13.13%		13.85%		0.400
Mechanical ventilation > 48 h, %	2.00%		2.00%		0.941
Length of hospital stay, d, mean / SD	12.34d	7.71d	17.75d	10.64d	< 0.001
Reimbursement, €, mean / SD	€28,723	€5364	€29,917	€6773	< 0.001

N: number of procedures; SD: standard deviation.

p-values based on chi-square test or t-test, as appropriate.

### Endpoint emergency admission

**Figure 1:**
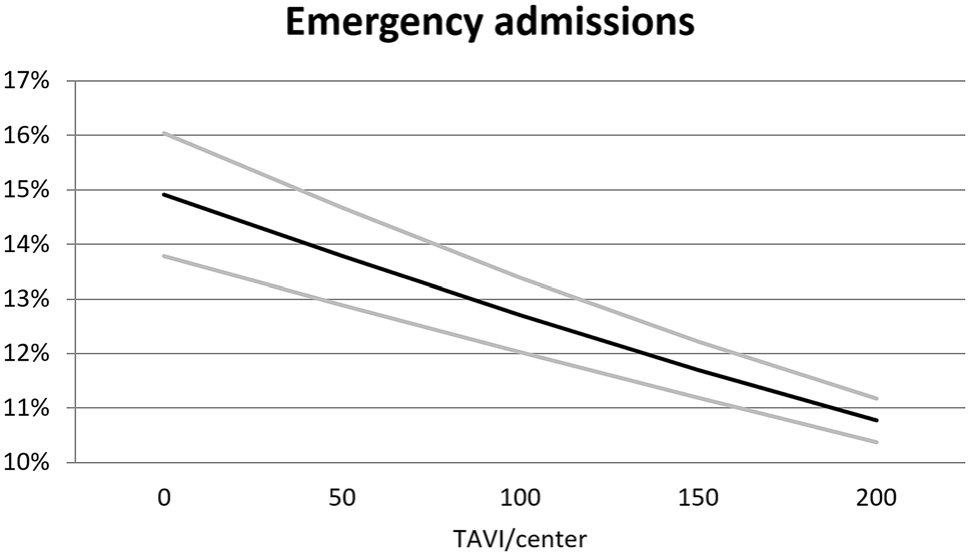
Proportion of emergency admissions in all TAVI interventions per center in Germany in 2018. Predicted emergency admissions (black line) and 95% confidence intervals (grey lines), divided up according to the total number of TAVI cases treated per hospital and year.

Regarding the proportion of emergency admissions in all TAVI interventions, divided up according to the total number of cases treated per hospital and year, lower volume centers treated relatively more emergency cases than higher volume centers (*p* < 0.001): In detail, centers conducting less than 50 TAVI procedures were associated with an emergency admission rate of ~ 15% while centers conducting more than 200 TAVI procedures had an emergency admission rate of ~ 11% (Fig. 1).

### Propensity score adjustment

**Figure 2:**
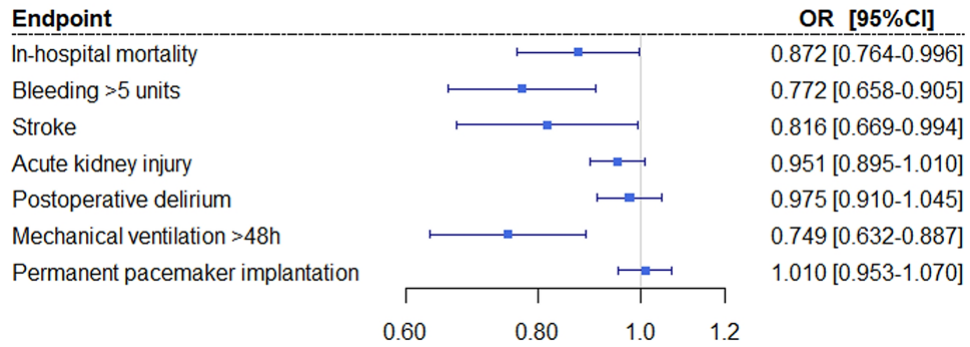
Predicted risk reduction associated with treatment in a higher volume center (per additional 10 emergency admissions per center). CI: confidence interval; OR: odds ratio.

**Figure 3:**
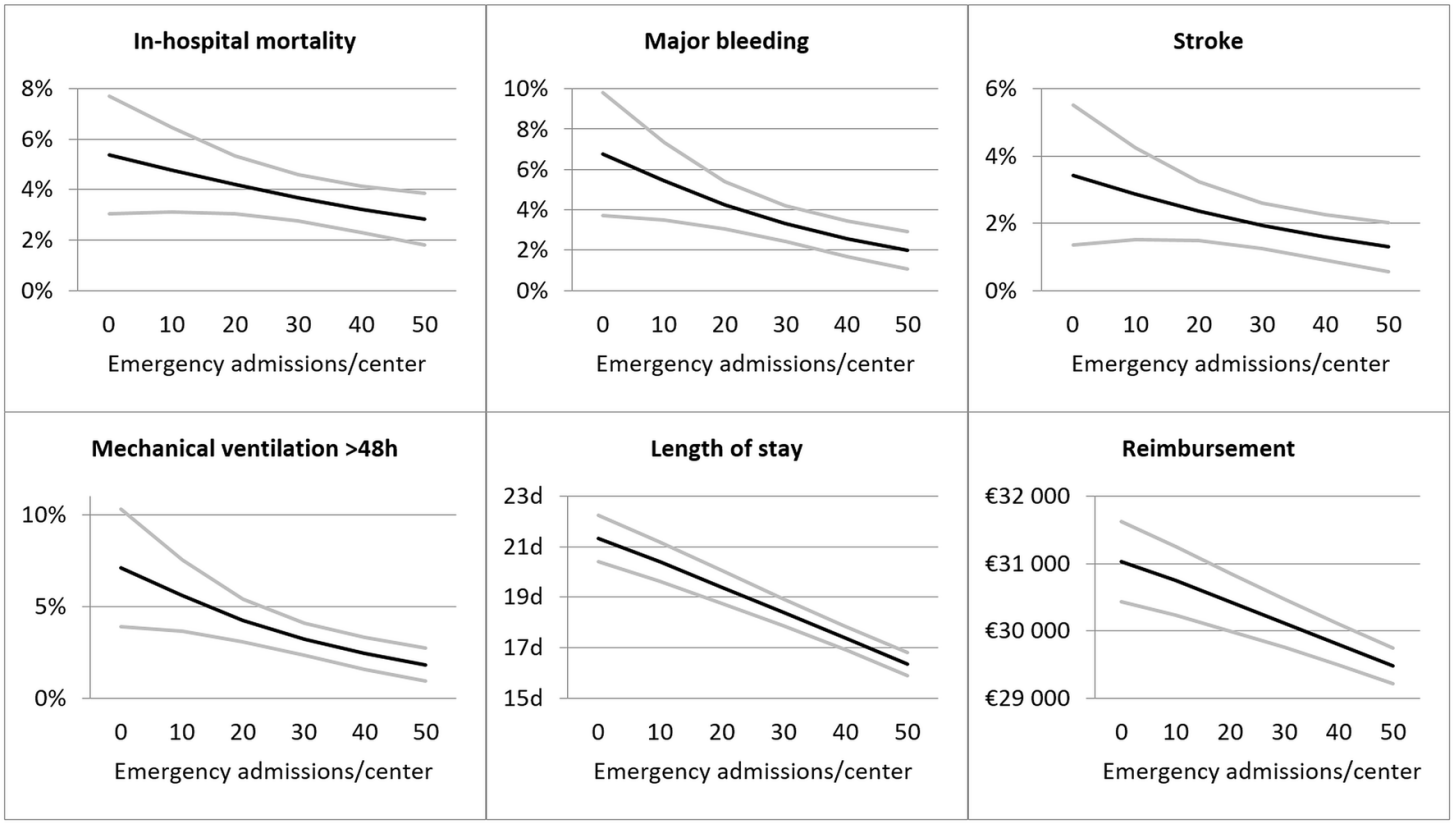
Continuous impact of TAVI procedure volumes with emergency admission in Germany in 2018. Presentation of significant factors. Predicted outcome (black line) and 95% confidence intervals (grey lines), divided up according to the number of TAVI cases with emergency admission treated per hospital and year. For the complete data see Supplementary Table 1.

When analyzing the outcomes for an increase in volume per 10 emergency admissions, after propensity score adjustment, higher volume centers showed significantly better outcomes vs lower volume centers for in-hospital mortality (OR = 0.872, *p* = 0.043), major bleeding (OR = 0.772, *p* = 0.001), stroke (OR = 0.816, *p* = 0.044), mechanical ventilation > 48 h (OR = 0.749, *p* = 0.001), length of hospital stay (risk adjusted difference in days of hospitalization per 10 emergency admissions: − 1.01 days, *p* < 0.001), and reimbursement (risk adjusted difference in reimbursement per 10 emergency admissions: -€314.89, *p* < 0.001; Figs. 2, 3, Supplementary Table 1). No relationship was seen in acute kidney injury (OR = 0.951, *p* = 0.104), postoperative delirium (OR = 0.975, *p* = 0.480), and permanent pacemaker implantation (OR = 1.010, *p* = 0.732).

## Discussion

We examined all 17,295 balloon-expandable and self-expanding transfemoral TAVI in Germany in 2018 and extracted those patients with emergency admission. Our study shows that lower volume centers treat relatively more emergency cases than higher volume centers in transfemoral TAVI in Germany, but higher volume centers provide significantly better results both in terms of in-hospital mortality and complication rates as well as resource utilization parameters.

Regarding baseline characteristics, it seems logical that emergency patients have a higher EuroSCORE and a comparatively higher heart failure class NYHA III/IV, since emergency interventions inherently cannot be planned and take place when the patient is in particularly poor health. Accordingly, a higher proportion of pre-existing disease is understandable, since such patients are usually sicker and an emergency occurs more often. This is also reflected in the outcomes, which are mainly worse in emergencies than in all patients who mostly received elective intervention.

Bansal et al.^16^ also investigate in a recent article the influence of annual hospital volumes on the amount and the outcome of urgent and emergent TAVI in the United States. The authors see that higher volume centers perform more urgent and emergent TAVI. This is in contrast to our findings. In Germany, emergent TAVI procedures are carried out relatively more frequently in lower volume centers, while higher volume centers do more elective interventions. However, it should be noted: Lower volume centers treat more emergencies as a percentage (~ 15 vs 11%) but far fewer cases in absolute terms, since nowadays TAVI in Germany is mostly conducted in higher volume centers^11^. So lower volume centers treat only a few emergency cases in absolute terms. Nevertheless, in such emergencies they play a decisive role in the medical care structure in Germany, since smaller centers are often the closest centers available. However, the outcomes in this article and our German data point in the same direction: Bansal et al.^16^ report on better results in centers with higher volume of urgent and emergent TAVI procedures for in-hospital mortality, stoke, acute kidney injury, vascular complications, and length of stay which is also mostly reflected in our results.

It can be assumed that the outcomes will continue to improve in the future as the centers and interventionalists become even more experienced and the intervention becomes even safer.

### Limitations

Our study has certain limitations beyond those typical of retrospective studies, in accordance with previous analyses^11,12^. First, it relies on administrative data, so coding errors are almost unavoidable. Usually, however, 20% of DRG are reviewed by independent physician teams from health insurances, so overall reliability should be good. Furthermore, risk adjustment included parameters whose reliability can’t be fully secured, and we can’t guarantee that all parameters of relevance are included in the model. For example, no information is available in the dataset on the exact types of valves or devices, the left ventricular ejection fraction, mean gradients, aortic valve area, or the distinction between native valve and valve-in-valve procedures. In addition, we can only compare hospitals in Germany, but not interventionalists. Therefore, no statements about volume-outcome relationships at the level of interventionalists are possible. In addition, due to the coding, it is ultimately not possible to differentiate whether the reason for the emergency admission was primarily the aortic valve stenosis or another reason. Nevertheless, it can be strongly assumed that a correspondingly severe aortic valve stenosis with the need for TAVI in the same hospital stay was a decisive point in the majority of admissions. Finally, no long-term follow-up is possible because the data source used does not allow a connection between different hospital stays of the same patient. Our study thus solely provides data on in-hospital outcomes, although for a very large, complete national yearly cohort of procedures.

## Conclusions

In summary, we examined a nationwide cohort with over 17,000 balloon-expandable or self-expanding transfemoral TAVI in Germany in 2018 with a focus on about 1700 patients with emergency admission. Data show that lower volume centers treat relatively more emergency cases than higher volume centers in transfemoral TAVI in Germany, but higher volume centers provide significantly better outcomes both in terms of in-hospital mortality and complication rates as well as resource utilization parameters.

## Supplementary Information

Supplementary Information.

## Data Availability

Data are available upon reasonable request (contact: Dr. Klaus Kaier, kaier@imbi.uni-freiburg.de). The patients’ data are stored on the server of the Federal Bureau of statistics and are not available due to data protection. The calculated raw data are sent anonymised to the scientist.
