# Supplementary material for: Hospital intervention volume affects outcomes of emergency transcatheter aortic valve implantations in Germany
Source: Sci Rep. 2022 Oct 19;12:17483. doi: 10.1038/s41598-022-20336-y (PMC9581927; doi:10.1038/s41598-022-20336-y)
Supplement: Supplementary file 1 — Supplementary Information. [file 41598_2022_20336_MOESM1_ESM.pdf]

Hospital intervention volume affects outcomes of emergency transcatheter aortic valve implantations in Germany

Supplementary material

Vera Oettinger, MD, M.Sc.; Adrian Heidenreich, MD; Klaus Kaier, PhD;

Manfred Zehender, MD, PhD; Christoph Bode, MD; Daniel Duerschmied, MD;

Constantin von zur Mühlen, MD; Dirk Westermann, MD; Peter Stachon, MD

Corresponding author:

Dr. Vera Oettinger, M.Sc.

Department of Cardiology and Angiology, University Heart Center

Medical Center – University of Freiburg

Hugstetter Str. 55

79106 Freiburg, Germany

vera.oettinger@uniklinik-freiburg.de

Supplementary Table 1: Propensity score adjustment for in-hospital outcomes of patients with balloon-expandable or self-expanding TAVI in Germany in 2018

Number of emergeny cases per center	In-hospital mortality				Bleeding >5 units				Stroke				Acute kidney injury				Delirium				Mechanical ventilation >48h				Permanent pacemaker implantation				Length of hospital stay				Reimbursement			
	**OR**	**p-value**	**95% CI**		**OR**	**p-value**	**95% CI**		**OR**	**p-value**	**95% CI**		**OR**	**p-value**	**95% CI**		**OR**	**p-value**	**95% CI**		**OR**	**p-value**	**95% CI**		**OR**	**p-value**	**95% CI**		**Coeff [d]**	**p-value**	**95% CI**		**Coeff [€]**	**p-value**	**95% CI**	
**Increase per 10 emergency cases**	0.872	**0.043**	0.764	0.996	0.772	**0.001**	0.658	0.905	0.816	**0.044**	0.669	0.994	0.951	**0.104**	0.895	1.010	0.975	**0.480**	0.910	1.045	0.749	**0.001**	0.632	0.887	1.010	**0.732**	0.953	1.070	-1.01	**0.000**	-1.19	-0.84	-314.89	**0.000**	-412.98	-216.80
**Predicted rates or means**																																				
**0**	0.054		0.030	0.077	0.068		0.037	0.098	0.034		0.014	0.055	0.173		0.139	0.207	0.109		0.082	0.137	0.071		0.039	0.103	0.134		0.105	0.163	21.327		20.420	22.233	31028.150		30435.920	31620.370
**10**	0.048		0.031	0.065	0.054		0.035	0.073	0.029		0.015	0.042	0.167		0.140	0.194	0.107		0.085	0.129	0.056		0.036	0.075	0.135		0.111	0.160	20.415		19.639	21.191	30744.750		30230.240	31259.250
**20**	0.042		0.030	0.054	0.042		0.031	0.054	0.024		0.015	0.032	0.160		0.139	0.181	0.105		0.087	0.122	0.042		0.031	0.054	0.137		0.117	0.156	19.402		18.757	20.046	30429.860		29996.940	30862.780
**30**	0.037		0.028	0.046	0.033		0.024	0.042	0.019		0.013	0.026	0.153		0.136	0.171	0.102		0.087	0.117	0.032		0.023	0.041	0.138		0.121	0.155	18.389		17.852	18.926	30114.970		29755.300	30474.650
**40**	0.032		0.023	0.041	0.026		0.017	0.034	0.016		0.009	0.023	0.147		0.130	0.164	0.100		0.085	0.115	0.024		0.015	0.033	0.139		0.122	0.156	17.376		16.904	17.847	29800.090		29499.150	30101.020
**50**	0.028		0.018	0.038	0.020		0.011	0.029	0.013		0.006	0.020	0.141		0.121	0.161	0.098		0.081	0.115	0.018		0.009	0.027	0.140		0.121	0.159	16.362		15.897	16.828	29485.200		29218.740	29751.660

CI: confidence interval; OR: odds ratio
